# Dolutegravir/Lamivudine Is Noninferior to Continuing Dolutegravir- and Non-Dolutegravir-Based Triple-Drug Antiretroviral Therapy in Virologically Suppressed People With Human Immunodeficiency Virus: DUALING Prospective Nationwide Matched Cohort Study

**DOI:** 10.1093/ofid/ofae160

**Published:** 2024-03-18

**Authors:** Marta Vasylyev, Ferdinand W N M Wit, Carlijn C E Jordans, Robin Soetekouw, Steven F L van Lelyveld, Gert-Jan Kootstra, Corine E Delsing, Heidi S M Ammerlaan, Marjo E E van Kasteren, Annemarie E Brouwer, Eliane M S Leyten, Mark A A Claassen, Robert-Jan Hassing, Jan G den Hollander, Marcel van den Berge, Anna H E Roukens, Wouter F W Bierman, Paul H P Groeneveld, Selwyn H Lowe, Berend J van Welzen, Olivier Richel, Jeannine F Nellen, Guido E L van den Berk, Marc van der Valk, Bart J A Rijnders, Casper Rokx

**Affiliations:** Section of Infectious Diseases, Department of Internal Medicine, and Department of Medical Microbiology and Infectious Diseases, Erasmus University Medical Center, Rotterdam, The Netherlands; Stichting HIV Monitoring, Amsterdam, The Netherlands; Section of Infectious Diseases, Department of Internal Medicine, and Department of Medical Microbiology and Infectious Diseases, Erasmus University Medical Center, Rotterdam, The Netherlands; Department of Internal Medicine, Spaarne Gasthuis, Haarlem/Hoofddorp, The Netherlands; Department of Internal Medicine, Spaarne Gasthuis, Haarlem/Hoofddorp, The Netherlands; Department of Internal Medicine, Medisch Spectrum Twente, Enschede, The Netherlands; Department of Internal Medicine, Medisch Spectrum Twente, Enschede, The Netherlands; Department of Internal Medicine, Catharina Ziekenhuis Eindhoven, Eindhoven, The Netherlands; Department of Internal Medicine, Elisabeth Tweesteden Ziekenhuis, Tilburg, The Netherlands; Department of Internal Medicine, Elisabeth Tweesteden Ziekenhuis, Tilburg, The Netherlands; Department of Internal Medicine, Haaglanden Medisch Centrum, The Hague, The Netherlands; Department of Internal Medicine, Rijnstate Ziekenhuis, Arnhem, The Netherlands; Department of Internal Medicine, Rijnstate Ziekenhuis, Arnhem, The Netherlands; Department of Internal Medicine, Maasstadziekenhuis, Rotterdam, The Netherlands; Department of Internal Medicine, Admiraal de Ruyter Ziekenhuis, Vlissingen, The Netherlands; Department of Infectious Diseases, Leiden University Medical Center, Leiden, The Netherlands; Section of Infectious Diseases, Department of Internal Medicine, University of Groningen, University Medical Center Groningen, Groningen, The Netherlands; Department of Internal Medicine, Isala, Zwolle, The Netherlands; Infectious Diseases and Infection Prevention, Department of Internal Medicine and Department of Medical Microbiology, Maastricht University Medical Center, Maastricht, The Netherlands; Department of Internal Medicine, University Medical Center Utrecht, Utrecht, The Netherlands; Section of Infectious Diseases, Department of Internal Medicine, Radboud University Medical Center, Nijmegen, The Netherlands; Amsterdam Infection and Immunity Institute, Department of Infectious Diseases, Amsterdam University Medical Centers, University of Amsterdam, Amsterdam, The Netherlands; Department of Internal Medicine, Onze Lieve Vrouwe Gasthuis, Amsterdam, The Netherlands; Stichting HIV Monitoring, Amsterdam, The Netherlands; Amsterdam Infection and Immunity Institute, Department of Infectious Diseases, Amsterdam University Medical Centers, University of Amsterdam, Amsterdam, The Netherlands; Section of Infectious Diseases, Department of Internal Medicine, and Department of Medical Microbiology and Infectious Diseases, Erasmus University Medical Center, Rotterdam, The Netherlands; Section of Infectious Diseases, Department of Internal Medicine, and Department of Medical Microbiology and Infectious Diseases, Erasmus University Medical Center, Rotterdam, The Netherlands

**Keywords:** 2DR, dolutegravir, HIV, lamivudine, real-world, virological failure

## Abstract

**Background:**

Confirming the efficacy of dolutegravir/lamivudine in clinical practice solidifies recommendations on its use.

**Methods:**

Prospective cohort study (DUALING) in 24 human immunodeficiency virus (HIV) treatment centers in the Netherlands. HIV RNA–suppressed cases were on triple-drug antiretroviral regimens without prior virological failure or resistance and started dolutegravir/lamivudine. Cases were 1:2 matched to controls on triple-drug antiretroviral regimens by the use of dolutegravir-based regimens, age, sex, transmission route, CD4^+^ T-cell nadir, and HIV RNA zenith. The primary endpoint was the treatment failure rate in cases versus controls at 1 year by intention-to-treat and on-treatment analyses with 5% noninferiority margin.

**Results:**

The 2040 participants were 680 cases and 1380 controls. Treatment failure in the 390 dolutegravir-based cases versus controls occurred in 8.72% and 12.50% (difference: −3.78% [95% confidence interval {CI}, −7.49% to .08%]) by intention-to-treat and 1.39% and 0.80% (difference: 0.59% [95% CI, –.80% to 1.98%]) by on-treatment analyses. The treatment failure risk in 290 non-dolutegravir-based cases was also noninferior to controls. Antiretroviral regimen modifications unrelated to virological failure explained the higher treatment failure rate by intention-to-treat. A shorter time on triple-drug antiretroviral therapy and being of non-Western origin was associated with treatment failure. Treatment failure, defined as 2 consecutive HIV RNA >50 copies/mL, occurred in 4 cases and 5 controls but without genotypic resistance detected. Viral blips occured comparable in cases and controls but cases gained more weight, especially when tenofovir-based regimens were discontinued.

**Conclusions:**

In routine care, dolutegravir/lamivudine was noninferior to continuing triple-drug antiretroviral regimens after 1 year, supporting the use of dolutegravir/lamivudine in clinical practice.

**Clinical Trials Registration:**

NCT04707326.

## Teaser

DUALING is a nationwide study in the Netherlands that provides robust empirical evidence from routine clinical care that virologically suppressed people with HIV-1 on triple-drug antiretroviral regimens can safely switch to dolutegravir/lamivudine.

Significant advancements in antiretroviral treatment (ART) transformed an infection with the human immunodeficiency virus (HIV) into a chronic condition. Since ART is lifelong, reducing the antiretroviral drug load is appealing from a toxicity prevention perspective, provided that ongoing viral suppression is guaranteed. Until recently, triple-drug ART was standard of care. This changed when 2-drug regimens (2DR), initially with boosted protease inhibitors, demonstrated acceptable effectiveness when used in virologically suppressed populations [1]. Side-effect profiles, the need for multiple pills, the lower efficacy when combined with raltegravir, and the amenability for drug interactions limited its use in clinical practice. The introduction of a dolutegravir (DTG)–based 2DR including lamivudine (3TC) overcame most of these limitations. This combination maintained viral suppression in registration trials [2, 3], which enabled the uptake of DTG-based 2DR in treatment guidelines [4–6]. Many virologically suppressed people with HIV (PWH) switched their triple-drug ART following the roll-out of DTG/3TC in clinical practice. Clinical monitoring of this group is necessary to timely identify suboptimal treatment outcomes with DTG/3TC outside controlled trial settings and in populations less represented there.

Subsequently, several cohort studies evaluated the outcomes of using DTG/3TC in routine clinical care in PWH on suppressive triple-drug ART [7–23]. These studies were conducted in a variety of settings, mostly in Europe. Overall, the results indicated sustained virological suppression with DTG/3TC.

Despite favorable signals, these studies also had relevant methodological limitations that left uncertainty on the exact potency of DTG/3TC when used in virologically suppressed PWH in routine care. The most important limitation was the lack of a matched comparator arm of PWH who continued their triple-drug ART. Also, most studies were retrospective by design, contained mixed study populations with different 2DR, or also included ART-naive PWH. These aspects complicated isolating the effectiveness of DTG/3TC in clinical practice. Only 1 cohort (Antiretroviral Resistance Cohort Analysis database (ARCA)) on off-label DTG/3TC use in PWH with resistance had matched controls [13]. This study pooled PWH on DTG/3TC and DTG with rilpivirine without disaggregating the treatment outcomes to DTG/3TC use. An optimal evaluation in the routine clinical practice where PWH on a triple-drug ART switch to DTG/3TC instead of continuing their triple-drug antiretroviral regimen thus remains to be done.

The aim of the dual-drug integrase inhibitor–based therapy (DUALING) study was to assess the treatment outcomes of starting DTG/3TC compared to continuing triple-drug ART in virologically suppressed PWH in the nationwide AIDS Therapy Evaluation in the Netherlands (ATHENA) cohort.

## METHODS

### Study Design and Population

DUALING is an ongoing prospective cohort study in the 24 HIV treatment centers in the Netherlands. Cases were virologically suppressed PWH who switched from a triple-drug regimen to DTG/3TC in routine care. Cases were identified at 9 of the 24 HIV treatment centers. These 9 centers had agreed upon implementing a proactive switch strategy from DTG-based triple-drug regimens to DTG/3TC using criteria described below. The cases from these centers were matched to controls treated at 1 of the other 15 treatment centers and who also fulfilled the switch criteria but had continued their triple-drug regimens. The decision to switch ART and all other medical decisions during follow-up in cases and controls were made by the treating physician in the routine care.

Cases were included at the start of DTG/3TC and were adults with HIV on triple-drug ART with a last plasma HIV RNA <50 copies/mL. A viral blip up to 200 copies/mL as last HIV RNA measurement before the start of DTG/3TC was allowed. We stratified cases into 2 groups according to the use of a DTG-based or non-DTG-based triple-drug ART. Prior virological failure (defined by any previous discontinued 3TC- or emtricitabine [FTC]–containing ART while the viral load was >1000 copies/mL), documented mutations associated with at least low-level 3TC or DTG resistance [24], and medication nonadherence documented by the treating physician in the prior 3 months were not allowed. Hepatitis B coinfection was allowed in controls only to reflect the treatment guidelines during the study. Cases were 1:2 matched to controls on the current use of DTG in triple-drug ART, age, sex, transmission route, CD4^+^ T-cell nadir below or above 200 cells/μL, and HIV RNA zenith below or above 100 000 copies/mL. Controls were required to have a clinical visit within a 3-month window around the DTG/3TC start date of the matching case. Follow-up in controls started at their clinical visit date closest in calendar time to the case DTG/3TC start date. This was to ensure that cases and controls were similarly affected by possible HIV treatment guideline changes over time and to other medical issues that could emerge as happened with the coronavirus disease 2019 pandemic. With >2 eligible controls, the clinical visit date closest to the DTG/3TC start date determined the selection of the controls. Cases and controls were clinically monitored half-yearly by their treating physician with HIV RNA measurements. Physicians were free to schedule an additional HIV RNA measurement 3 months after the switch.

### Outcomes

The primary endpoint was the risk difference in treatment failure rate between cases and controls in the DTG-based stratum at 1 year (window: −3 to +6 months) of follow-up by on-treatment (OT) and intention-to-treat (ITT) analyses. Treatment failure was defined as 2 consecutive HIV RNA measurements >50 copies/mL on the same ART or a single HIV RNA >50 copies/mL followed by loss to follow-up or a change in ART. The ITT and OT populations were predefined. In the OT population, those who were lost to follow-up or switched ART while HIV RNA was ≤50 copies/mL were not considered treatment failures. In the ITT population, these events were defined as treatment failure. Controls were censored when switching from triple-drug ART to DTG/3TC. Treatment success was defined as plasma HIV RNA <50 copies/mL at the 1-year timepoint. A single measurable HIV RNA >50 copies/mL at this timepoint without a switch in antiretroviral regimen did not fit the treatment failure criteria and was therefore categorized as treatment success at 1 year. PWH were considered lost to follow-up when no HIV RNA measurement was done for >52 weeks. The secondary endpoints were treatment failure rate in cases starting DTG/3TC from a non-DTG-based triple-drug regimen, the frequency of viral blips (detectable HIV RNA preceded and followed by HIV RNA <50 copies/mL), and emergent resistance mutations in cases and controls [24]. Subgroup analyses of treatment failure were included. A post hoc–defined analysis of weight change was done based on accumulating insights in the field during the study on ART-associated weight gain.

### Statistical Analysis

For the sample size calculation, we used a 0.95 viral suppression rate, a 1:2 case-to-control ratio, and an interclass correlation of 0.1. With a noninferiority margin δ = 0.05, 1-sided α = .05, the required sample size was 390 cases for 90% power to detect noninferiority. Continuous data were reported as means or medians and categorical data as percentages. Wilcoxon rank-sum tests, *t* tests, and χ^2^ tests were used. The risk difference in the treatment failure rate in cases versus controls was calculated as a point estimate with a 1-sided 90% confidence interval (CI) and a 2-sided 95% CI. The predefined upper limit of noninferiority was 5%. Noninferiority was concluded when the upper limit of the CI did not exceed this threshold. A logistic regression model was constructed to calculate adjusted odds ratios (ORs) on treatment failure in cases by sex, age, ethnicity, transmission route, CD4^+^ T-cell count (nadir and current), HIV RNA zenith, years on ART, and DTG-based triple-drug ART use.

### Patient Consent Statement

All participants provided consent for inclusion in ATHENA and the use of their data collected in the routine care. The ATHENA cohort was approved by the institutional review boards of all HIV treatment centers in the Netherlands who waived the need for written informed consent and approved an opt-out procedure after being informed of the purpose of the data collection. The study was registered at ClinicalTrials.gov (NCT04707326).

## RESULTS

### Baseline Characteristics

A total of 2040 PWH were included up to December 2020 (680 DTG/3TC cases and 1360 controls). The 680 selected cases came from 7544 eligible PWH in care at the 9 HIV treatment centers used for case selection. The clinical characteristics of selected cases and the eligible population that was not selected were comparable within and between sites that provided cases and controls (Supplementary Data). Of cases, 390 were on DTG-based triple-drug ART and 290 had non-DTG-based triple-drug ART before starting DTG/3TC. Baseline characteristics of the cases and controls were well balanced and in most controls a viral load had been measured in a 7-day window around the date of switch to DTG/3TC of the matched case (Table 1). Overall, cases and controls were males (84.3%) of Western origin with HIV acquired most frequently through sexual contact with men. Cases had a shorter time on ART. Current and nadir CD4^+^ T-cell counts and HIV RNA zenith were comparable. The most frequently used DTG-based triple-drug ART was a single-tablet regimen with 3TC and abacavir. For those not on DTG, nonnucleoside reverse transcriptase inhibitors were most frequently used with a tenofovir-containing nucleoside reverse transcriptase inhibitor backbone with FTC. Comorbidities were largely comparable across participants and strata, except for proportionally more hypertension in cases. A minority of cases and controls had a viral blip before starting DTG/3TC.

**Table 1.** Baseline Characteristics of the DUALING Study Population

Characteristic	DTG-Based ART		Non-DTG-Based ART	
	DTG/3TC (Cases) (n = 390)	DTG-Based Triple-Drug ART (Controls) (n = 780)	DTG/3TC (Cases) (n = 290)	Non-DTG-Based Triple-Drug ART (Controls) (n = 580)
Age, y, median (IQR)	48 (37–57)	48 (38–57)	50 (39–59)	50 (40–59)
Male sex	344 (88.2)	688 (88.2)	229 (79.0)	458 (79.0)
Region of birth				
Netherlands	264 (67.7)	471 (60.4)	170 (58.6)	344 (59.3)
Western	45 (11.5)	68 (8.7)	31 (10.7)	48 (8.3)
Sub-Saharan Africa	19 (4.9)	74 (9.5)	33 (11.4)	71 (12.2)
Latin America/Caribbean	34 (8.7)	100 (12.8)	35 (12.1)	79 (13.6)
South Asia	19 (4.9)	74 (9.5)	8 (2.8)	24 (4.1)
Other	9 (2.3)	34 (4.4)	13 (4.5)	14 (2.4)
HIV transmission route				
MSM	283 (72.6)	576 (73.8)	185 (63.8)	373 (64.3)
Heterosexual	80 (20.5)	170 (21.8)	88 (30.3)	183 (31.6)
Intravenous drug use	3 (0.8)	2 (0.3)	2 (0.7)	4 (0.7)
Blood–blood contact	2 (0.5)	2 (0.3)	4 (1.4)	2 (0.3)
Vertical	3 (0.8)	5 (0.6)	1 (0.3)	0 (0.0)
Unknown	19 (4.9)	25 (3.2)	10 (3.4)	18 (3.1)
Prior ART use, y, median (IQR)	5 (3–9)	8 (5–12)	9 (6–14)	11 (8–15)
CD4^+^ T-cell count nadir, cells/μL, median (IQR)	330 (200–485)	310 (190–471)	290 (180–437)	270 (170–380)
HIV RNA zenith, log_10_ copies/mL, median (IQR)	4.9 (4.4–5.3)	4.8 (4.3–5.4)	5.0 (4.4–5.3)	5.0 (4.5–5.4)
Prior AIDS diagnosis	52 (13.3)	138 (17.7)	31 (10.7)	96 (16.6)
Current CD4^+^ T-cell count, cells/μL, median (IQR)	690 (500–890)	736 (561–934)	750 (540–950)	730 (569–930)
HIV RNA 50–200 copies/mL (blip) at start of follow-up	8 (2.1)	11 (1.4)	3 (1.0)	5 (0.9)
No. of prior ART regimens, median (IQR)	2 (1–3)	2 (1–3)	2 (1–3)	2 (1–3)
NRTI backbone				
3TC	390 (100)	0 (0)	290 (100)	0 (0)
ABC/3TC	0 (0)	600 (76.9)	0 (0)	28 (4.3)
TDF/FTC	0 (0)	105 (13.5)	0 (0)	233 (40.2)
TDF/3TC	0 (0)	1 (0.1)	0 (0)	49 (8.4)
TAF/FTC	0 (0)	74 (9.5)	0 (0)	269 (46.4)
TAF/3TC	0 (0)	0 (0)	0 (0)	1 (0.1)
Anchor drug class				
INSTI	390 (100)	780 (100)	290 (100)	182 (31.4)
NNRTI	0 (0)	0 (0)	0 (0)	332 (57.2)
PI	0 (0)	0 (0)	0 (0)	66 (11.4)
Reasons to switch to DTG/3TC				
ART simplification	216 (55.4)	…	109 (37.6)	…
New treatment option	96 (24.6)	…	25 (8.6)	…
Toxicity	56 (14.4)	…	126 (43.5)	…
Drug–drug interaction	0 (0)	…	10 (3.4)	…
Other	13 (3.3)	…	13 (4.5)	…
Unknown	9 (2.3)	…	7 (2.4)	…
Comorbidities				
Hepatitis B antigen positive	0 (0)	24 (3.1)	0 (0)	27 (4.7)
Hepatitis C IgG positive	28 (7.2)	70 (9.0)	27 (9.3)	53 (9.1)
Obesity	44 (11.3)	93 (11.9)	34 (11.7)	69 (11.9)
Diabetes mellitus type 2	12 (3.1)	24 (3.1)	16 (5.5)	29 (5.0)
Hypertension	141 (36.2)	245 (31.4)	94 (32.4)	151 (26.0)
Chronic kidney disease	42 (10.8)	92 (11.8)	17 (5.9)	33 (5.7)
Cardiovascular disease	10 (2.6)	25 (3.2)	12 (4.1)	25 (4.3)
Stroke	4 (1.0)	8 (1.0)	3 (1.0)	8 (1.4)
Non-AIDS malignancy	15 (3.8)	29 (3.7)	9 (3.1)	23 (4.0)

Data are presented as No. (%) unless otherwise indicated.

Abbreviations: 3TC, lamivudine; ABC, abacavir; ART, antiretroviral therapy; DTG, dolutegravir; FTC, emtricitabine; HIV, human immunodeficiency virus; IgG, immunoglobulin G; INSTI, integrase strand transfer inhibitor; IQR, interquartile range; MSM, men who have sex with men; NNRTI, nonnucleoside reverse transcriptase inhibitor; NRTI, nucleoside reverse transcriptase inhibitor; PI, protease inhibitor; TAF, tenofovir alafenamide; TDF, tenofovir disoproxil fumarate.

### Treatment Outcomes

#### DTG-Based Cases Versus Controls

**Figure 1.**
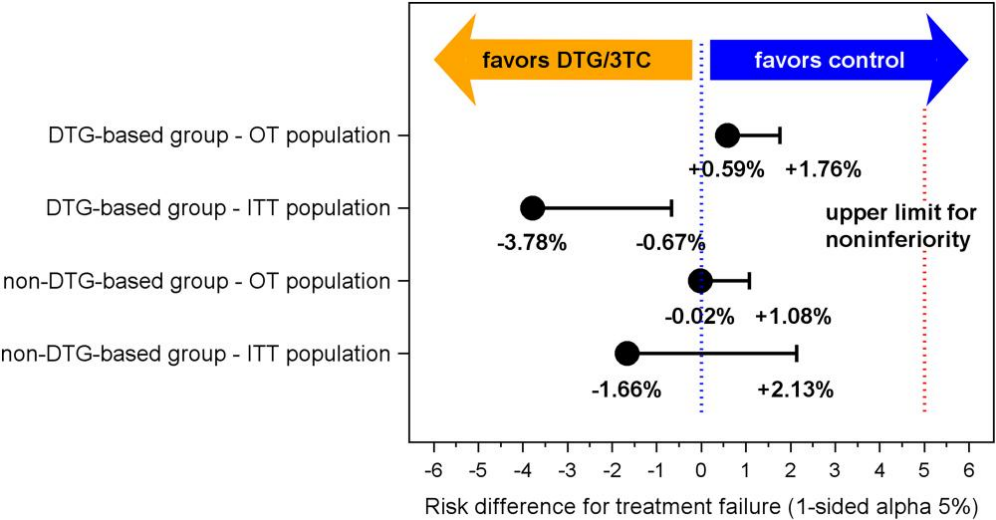
Treatment failure risk difference with 1-sided 90% confidence interval (CI) between cases on dolutegravir/lamivudine and controls on triple-drug antiretroviral regimens. Treatment failure risk difference with 1-sided 90% CI at 1 year of follow-up between cases and controls who were virologically suppressed on either a dolutegravir- or a non-dolutegravir-containing triple-drug antiretroviral regimen and started dolutegravir/lamivudine or continued triple-drug antiretroviral regimens by on-treatment and intention-to-treat analysis. The vertical red dotted line indicates the 5% noninferiority margin. Abbreviations: 3TC, lamivudine; DTG, dolutegravir; OT, on-treatment; ITT, intention-to-treat.

**Figure 2.**
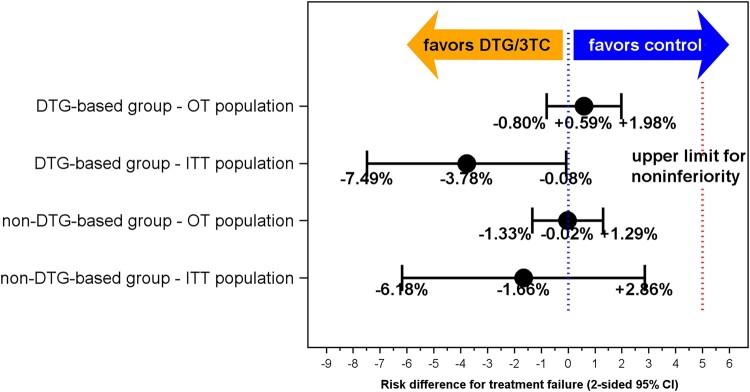
Treatment failure risk difference with 2-sided 95% confidence interval (CI) between cases on dolutegravir/lamivudine and controls on triple-drug antiretroviral regimens. Treatment failure risk difference with 2-sided 95% CI at 1 year of follow-up between cases and controls who were virologically suppressed on either a dolutegravir- or a non-dolutegravir-containing triple-drug antiretroviral regimen and started dolutegravir/lamivudine or continued triple-drug antiretroviral regimens by on-treatment and intention-to-treat analysis. The vertical red dotted line indicates the 5% noninferiority margin. Abbreviations: 3TC, lamivudine; CI, confidence interval; DTG, dolutegravir; OT, on-treatment; ITT, intention-to-treat.

In the OT population of the DTG-based stratum, the treatment failure rate after 1 year was comparable in cases and controls (1.39% and 0.80%; Table 2). The risk difference was 0.59% with a 90% CI upper limit of 1.76% (Figure 1) and a 95% CI of −.80% to 1.98% (Figure 2). The analysis in the ITT population indicated a somewhat higher treatment failure rate in controls than in cases (12.50% and 8.72%, respectively). The risk difference was −3.78% with a 90% CI upper limit of −.67% and a 95% CI of −7.49% to −.08% (Figures 1 and 2). The 5% noninferiority margin was not exceeded in any of these analyses, which confirmed the noninferiority of DTG/3TC. The OT population consisted of 361 cases and 628 controls and the ITT population of 390 cases and 712 controls. In the ITT analysis, 29 cases had treatment failure due to loss to follow-up or ART changes while having an HIV RNA <50 copies/mL. Of the 143 controls who switched ART while having an HIV RNA <50 copies/mL, 68 switched to DTG/3TC and were censored in the ITT analysis. During follow-up, 5 cases and 5 controls (OT population) and 34 cases and 89 controls (ITT population) had protocol-defined treatment failure.

**Table 2.** Treatment Outcomes at 1 Year of Follow-up in Cases on Dolutegravir/Lamivudine and Controls on Triple-Drug Antiretroviral Regimens in the On-Treatment and Intention-to-Treat Populations

Outcome	DTG-Based ART		Non-DTG-Based ART	
	DTG/3TC (Cases) (n = 390)	DTG-Based Triple-Drug ART (Controls) (n = 780)	DTG/3TC (Cases) (n = 290)	Non-DTG-Based Triple-Drug ART (Controls) (n = 580)
OT population: outcome at 1 year				
Censored: insufficient or lost to follow-up, pVL ≤50 at last contact	9 (2.31)	9 (1.15)	9 (3.10)	26 (4.48)
Failure: insufficient or lost to follow-up, pVL >50 at last contact	0 (0)	0 (0)	0 (0)	1 (0.17)
Censored: switched ART regimen, pVL ≤50 before switch	20 (5.13)	143 (18.33)	21 (7.24)	48 (8.28)
Failure: switched ART regimen, pVL >50 before switch	1 (0.26)	2 (0.26)	2 (0.69)	1 (0.17)
Failure: 2× pVL >50	4 (1.03)	3 (0.38)	0 (0)	2 (0.34)
Success: pVL ≤50	354 (90.77)	619 (79.36)	257 (88.62)	493 (85.00)
Success: single pVL >50 at the year 1 visit	2 (0.51)	4 (0.51)	1 (0.34)	9 (1.55)
OT population: outcome (dichotomized, excluding those censored)				
Treatment success	356 (98.61)	623 (99.20)	258 (99.23)	502 (99.21)
Treatment failure	5 (1.39)	5 (0.80)	2 (0.77)	4 (0.79)
Censored	29	152	30	74
ITT population: outcome at 1 year				
Failure: insufficient or lost to follow-up, pVL ≤50 at last contact	9 (2.31)	9 (1.15)	9 (3.10)	26 (4.48)
Failure: insufficient or lost to follow-up, pVL >50 at last contact	0 (0)	0 (0)	0 (0)	1 (0.17)
Failure: switched ART regimen, pVL ≤50 before switch	20 (5.13)	75 (9.62)	21 (7.24)	43 (7.41)
Censored: switched to DTG/3TC, pVL ≤50	0 (0)	68 (8.72)	0 (0)	5 (0.86)
Failure: switched ART regimen, pVL >50 before switch	1 (0.26)	2 (0.26)	2 (0.69)	1 (0.17)
Failure: 2× pVL >50	4 (1.03)	3 (0.38)	0 (0)	2 (0.34)
Success: pVL ≤50	354 (90.77)	619 (79.36)	257 (88.62)	493 (85.00)
Success: single pVL >50 at the year 1 visit	2 (0.51)	4 (0.51)	1 (0.34)	9 (1.55)
ITT population: outcome (dichotomized, excluding those censored)				
Treatment success	356 (91.28)	623 (87.50)	258 (88.97)	502 (87.30)
Treatment failure	34 (8.72)	89 (12.50)	32 (11.03)	73 (12.70)
Censored	0	68	0	5

Data are presented as No. (%). All pVL values are copies/mL.

Abbreviations: 3TC, lamivudine; ART, antiretroviral therapy; DTG, dolutegravir; ITT, intention-to-treat; OT, on-treatment; pVL, plasma viral load.

#### Non-DTG-Based Cases Versus Controls

In the non-DTG-based stratum, the treatment failure rate was 0.77% and 0.79% by OT and 11.03% and 12.70% by ITT analyses in cases and controls, respectively (Table 2). In both the ITT and the OT analyses, the 90% CI upper limit and 95% CI around the risk difference did not exceed the noninferiority margin (Figures 1 and 2). The OT population consisted of 260 cases and 506 controls and the ITT population of 290 cases and 575 controls (Table 2). Thirty cases were lost to follow-up or switched ART while HIV RNA was <50 copies/mL. They were censored in the OT population and considered treatment failure in the ITT population. Of the 74 controls who switched ART or were lost to follow-up while having an HIV RNA <50 copies/mL, 5 switched to DTG/3TC and were censored in the ITT analysis. During follow-up, 2 cases and 4 controls (OT population) and 32 cases and 73 controls (ITT population) had protocol-defined treatment failure.

#### Specific Treatment Outcomes in Cases and Controls

**Figure 3.**
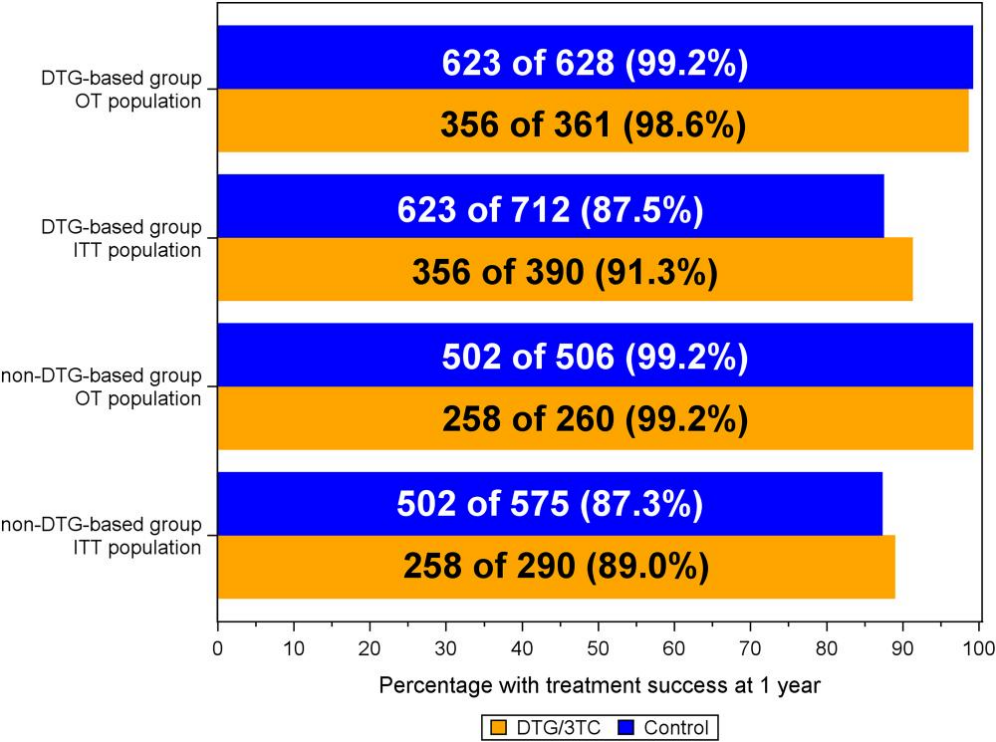
Treatment success rate in cases on dolutegravir/lamivudine and controls on triple-drug antiretroviral regimens. Treatment success rate at 1 year of follow-up in the on-treatment and intention-to-treat population between cases and controls who were virologically suppressed on either a dolutegravir- or a non-dolutegravir-containing triple-drug antiretroviral regimen and started dolutegravir/lamivudine or continued triple-drug antiretroviral regimens. Abbreviations: 3TC, lamivudine; DTG, dolutegravir; OT, on-treatment; ITT, intention-to-treat.

The treatment success rates were comparable for cases and controls in the strata (Figure 3) Upon further evaluation of 16 treatment failures in the OT population in both strata, 9 of them had 2 consecutive HIV RNA measurements >50 copies/mL. This included 4 cases and 3 controls in the DTG-based stratum and 2 controls in the non-DTG-based stratum (Table 2). Of these 9 participants, 2 controls had viremia >200 copies/mL (440 and 1120 copies/mL). The others subsequently resuppressed to <50 copies/mL or remained having low-level viremia <200 copies/mL without changing ART. A single control in the non-DTG-based stratum was lost to follow-up with a last HIV RNA >50 copies/mL. The other 6 of the 16 treatment failures (3 cases and 3 controls) consisted of a single HIV RNA between 50 and 200 copies/mL followed by a change in treatment regimen. ART modifications determined the majority of the protocol-defined treatment failures in the ITT analysis. No emergent genotypic mutations were identified in cases or controls during the observation period. Of the 16 PWH with a single last plasma HIV RNA >50 copies/mL at the end of the observation period, 11 had 51–200 copies/mL (3 cases) and 5 controls had HIV RNA >200 copies/mL. During follow-up, 1.6% of cases and 1.2% of controls had viral blips >50 copies/mL. The 27 PWH with a viral blip between 50 and 200 copies/mL at inclusion had more viral blips (22.2%) during follow-up compared to 3.0% of 2013 PWH without a viral blip at inclusion (Supplementary Data). These blips occurred at a fairly similar rate between cases and controls and the majority of blips were <200 copies/mL.

**Figure 4.**
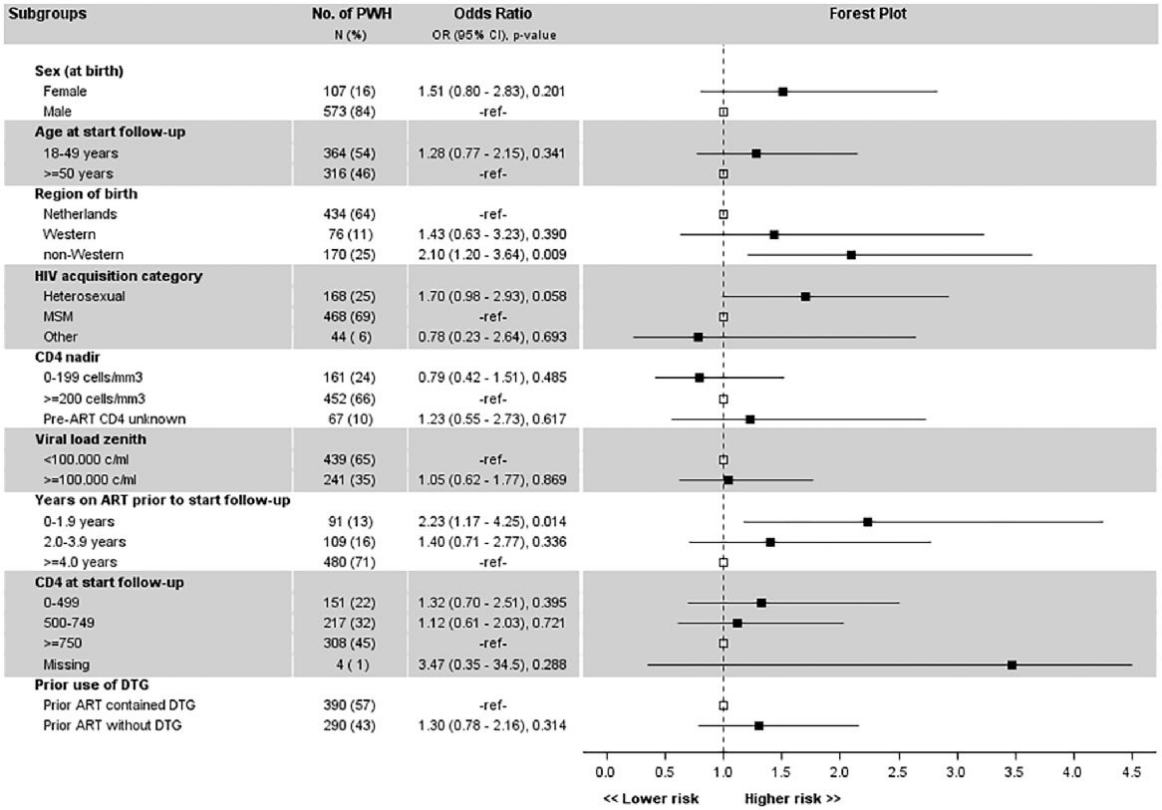
Subgroup analysis of treatment failure at 1 year of follow-up in 680 cases on dolutegravir/lamivudine. Square markers denote the odds ratio (OR). The vertical dashed line at OR = 1 is the line of no effect. Upper calipers are capped at value 4.5. The reference category uses open square markers at OR = 1. Abbreviations: 3TC, lamivudine; ART, antiretroviral therapy; CI, confidence interval; DTG, dolutegravir; HIV, human immunodeficiency virus; MSM, men who have sex with men; OR, odds ratio; PWH, people with human immunodeficiency virus.

**Figure 5.**
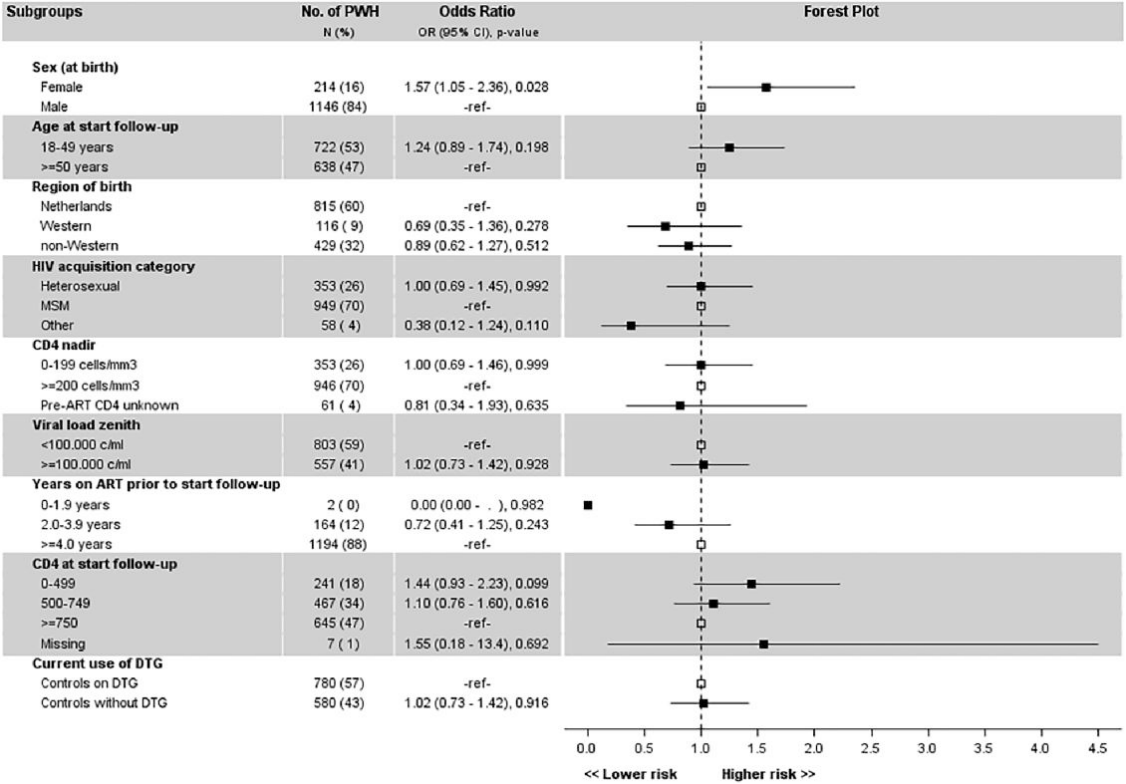
Subgroup analysis of treatment failure at 1 year of follow-up in 1380 controls continuing triple-drug antiretroviral therapy. Square markers denote the odds ratio (OR). The vertical dashed line at OR = 1 is the line of no effect. Upper calipers are capped at value 4.5. The reference category uses open square markers at OR = 1. Abbreviations: ART, antiretroviral therapy; CI, confidence interval; DTG, dolutegravir; HIV, human immunodeficiency virus; MSM, men who have sex with men; OR, odds ratio; PWH, people with human immunodeficiency virus.

The treatment failure rate in clinical subgroups from cases (n = 680) were analyzed by ITT (Figure 4). Treatment failure was associated with non-Western ethnicity (OR, 2.10 [95% CI, 1.20–3.64]) and time on ART of <2 years (OR, 2.23 [95% CI, 1.17–4.25]). Cases who were women or people with a heterosexual HIV acquisition category tended toward more treatment failure. No clear association with treatment failure was found for the other subgroups. When analyzing clinical subgroups from controls (n = 1360), women had more treatment failure (OR, 1.57 [95% CI, 1.05–2.36]) while too few controls had <2 years of ART exposure for a meaningful analysis (Figure 5). Further analyses of these subgroups indicated zero treatment failure in non-Western cases and controls in the OT analysis where the treatment failure rates were 1.72% versus 0.47% (risk difference: 1.25%, 90% CI upper limit 2.62%) in DTG-based and 1.08% versus 0.87% (risk difference: 0.21%, 90% CI upper limit 1.29%) in non-DTG-based cases and controls with a Western background. In a related subgroup analysis, none of the controls with hepatitis B had treatment failure (OT population), and excluding hepatitis B–coinfected controls did not impact treatment failure rates: risk differences of 0.57% (90% CI upper limit 1.74%) in the DTG-based group and of 0.06% (90% CI upper limit 1.06%) in the non-DTG-based group. Regarding women, the treatment failure rates were 2.50% versus 2.60% (risk difference: −0.10%, 90% CI upper limit 4.94%) in DTG-based and 0% versus 2.00% (risk difference: −2.00%, 90% CI upper limit +0.30%) in non-DTG-based female cases and controls (OT population).

Weight gain during follow-up was higher in cases (Supplementary Data). Compared to controls, the cases with weight registered before and after switching ART in the DTG-based stratum (n = 128) and the non-DTG-based stratum (n = 122) gained between 1.5 kg (*P* = .052) and 1.7 kg (*P* = .005) more weight per year compared to their matched controls, respectively. Weight gain was more prominent in the subgroup of cases that stopped a regimen containing tenofovir disoproxil fumarate (TDF) with a mean increase of 4.2 kg and 3.0 kg for DTG- and non-DTG-based cases, respectively, although especially the number of people who switched from a triple-drug regimen containing both DTG and TDF was small.

## DISCUSSION

The main conclusion from this study was that switching virologically suppressed PWH to DTG/3TC in routine care was noninferior to continuing triple-drug regimens 1 year after the switch both in ITT and OT analyses. This was independent of the type of ART regimen (DTG-based or not) at the start of DTG/3TC. True virological failure was rare in all groups. No emergent resistance mutations were found. Treatment outcomes on DTG/3TC were consistently favorable throughout subgroups. These outcomes reassure the findings from registration trials and substantiate the uptake of DTG/3TC in treatment guidelines.

DUALING is the largest controlled study done in routine clinical practice on DTG/3TC in virologically suppressed PWH and encompassed all HIV treatment centers in the Netherlands. Several aspects suggest the generalizability to other countries. We intentionally designed the study with well-matched controls on triple-drug ART to enhance the comparability of treatment outcomes in a clinical setting with trial results. This was a main limitation in previous studies on DTG/3TC after its market release. Second, as we used data from all 24 HIV treatment centers that exist in the Netherlands, we are confident that the results reflect clinical practice. Our findings are therefore likely applicable to other countries, especially those with comparable resources and demographical constitution of their HIV populations.

The observed treatment failure in virologically suppressed PWH on DTG/3TC was in line with prior estimates. From the randomized clinical trials, the open-label SALSA approximated routine clinical practice by allowing inclusion of all triple-drug ART [3]. This study concluded on noninferiority with DTG/3TC and saw very few treatment failures. Small numerical differences in the TANGO randomized clinical trial and 1 other cohort study might have suggested an association of treatment failure with lower CD4^+^ T-cell count [2, 21]. We did not find clear evidence for this, although, similar to these other studies, the majority had normal CD4^+^ T-cell counts. The data did confirm the association with a shorter duration on ART and treatment failure, as observed in some other cohorts [8–10, 22]. Similar to the Belgian study BREACH (Belgium Research on AIDS and HIV Consortium), a non-Western background was related to treatment failure. We also found more treatment failure in women, especially in the control group, of whom most were of non-Western origin. This observation could not be explained by a lower virological efficacy of DTG/3TC in these subgroups. Rather it can be a consequence of other factors not taken into account in this study, foremost socioeconomic or related to a patient's or care provider's behavior influencing the decision to switch therapy to DTG/3TC or subsequent treatment decisions. Adverse treatment outcomes in people from non-Western origins have been more frequently reported with other ART too [25].

The limitations of our study should be acknowledged. Despite matching, a nonrandomized design can never account for all unmeasured confounders. However, the known indicators for adverse treatment outcomes on DTG/3TC were included and balanced. We also ensured similar calendar time observation periods in matched case-control pairs. Still, minor differences between selected and nonselected individuals were observed, which could reflect different treatment practices between sites that provided the cases and controls. A selection bias favoring a switch to DTG/3TC in PWH who are expected to do well on DTG/3TC cannot be excluded. A longer follow-up is needed to conclude on long-term treatment outcomes. Follow-up of included PWH in DUALING is ongoing to provide these data. The inclusion reached the target number but could not prevent that some clinically relevant subgroups still remained small. In particular the number of PWH with low CD4^+^ T-cells <200 cells/μL was very small. More data from routine clinical practice with more immunocompromised PWH, or a future meta-analysis, are needed to evaluate the risk of virological failure in this population. The weight data should be interpreted cautiously since this endpoint was post hoc defined and therefore not systematically collected. However, the findings indicated possible increased weight gain after switching to DTG/3TC and, in line with other reports, suggested an influence of discontinuing TDF [21].

In conclusions, in this nationwide study from the Netherlands, switching virologically suppressed PWH to DTG/3TC was compared to PWH who continued a triple-drug ART and shown to be effective and safe in routine clinical care.

## Supplementary Data

Supplementary materials are available at *Open Forum Infectious Diseases* online. Consisting of data provided by the authors to benefit the reader, the posted materials are not copyedited and are the sole responsibility of the authors, so questions or comments should be addressed to the corresponding author.

## Supplementary Material

ofae160_Supplementary_Data

## Notes

***Author Contributions.*** Conceptualization: M. V., F. W., C. J., M. v. d. V., B. R., and C. R. Methodology: M. V., F. W., C. J., M. v. d. V., B. R., and C. R. Investigation: All authors. Visualization: M. V., F. W., and C. R. Funding acquisition: B. R. and C. R. Project administration: C. R. Supervision: F. W., M. v. d. V., B. R., and C. R. Writing—original draft: M. V., F. W., and C. R. Writing—review and editing: All authors.

***Acknowledgments.*** We would like to acknowledge Prof. Peter Reiss for his help in conceptualizing the study.

***Disclaimer.*** The funder did not have a role in data acquisition or analysis, nor in the decision to submit the data for publication.

***Financial support.*** The DUALING study was an investigator-initiated study supported by ViiV Healthcare (grant ID: 214246). The ATHENA database is maintained by Stichting HIV Monitoring and supported by a grant from the Dutch Ministry of Health, Welfare and Sport through the Center for Infectious Disease Control of the National Institute for Public Health and the Environment.
