# Supplementary material for: Dolutegravir/Lamivudine Is Noninferior to Continuing Dolutegravir- and Non-Dolutegravir-Based Triple-Drug Antiretroviral Therapy in Virologically Suppressed People With Human Immunodeficiency Virus: DUALING Prospective Nationwide Matched Cohort Study
Source: Open Forum Infect Dis. 2024 Mar 18;11(4):ofae160. doi: 10.1093/ofid/ofae160 (PMC10986854; doi:10.1093/ofid/ofae160)
Supplement: ofae160_Supplementary_Data [file ofae160_supplementary_data.docx]

**Supplementary data**

| **Supplementary table 1. Baseline characteristics of all non-participating eligible people with HIV in DUALING and non-DUALING sites** | | | | | |
| --- | --- | --- | --- | --- | --- |
|  | **DUALING sites** |  | **Non-DUALING sites** |  | |
|  | **Eligible DTG-based triple-drug ART but not switched to DTG/3TC**  **N=2178** | **Eligible non-DTG-based triple-drug ART but not switched to DTG/3TC**  **N=4686** | **Eligible DTG-based triple-drug ART but not selected as triple-drug ART control**  **N=1902** | **Eligible non-DTG-based triple-drug ART but not selected as triple-drug ART control**  **N=7828** | |
| **Age (median years, IQR)** | 48 (38-56) | 49 (40-57) | 49 (39-57) | 50 (42-58) | |
| **Male sex** | 1842 (84.6%) | 3750 (80.0%) | 1589 (83.5%) | 6633 (84.7%) | |
| **Region of birth** |  |  |  |  | |
| Netherlands | 1279 (59.0%) | 2831 (60.5%) | 1136 (60.0%) | 4591 (59.0%) | |
| Western | 110 (5.1%) | 214 (4.6%) | 92 (4.9%) | 551 (7.1%) | |
| Sub-Saharan Africa | 235 (10.8%) | 609 (13.0%) | 232 (12.3%) | 797 (10.2%) | |
| Latin America/Caribbean | 314 (14.5%) | 580 (12.4%) | 213 (11.3%) | 999 (12.8%) | |
| South Asia | 83 (3.8%) | 172 (3.7%) | 75 (4.0%) | 303 (3.9%) | |
| Other | 148 (6.8%) | 271 (5.8%) | 144 (7.6%) | 545 (7.0%) | |
| Missing | 9 | 9 | 10 | 42 | |
| **HIV transmission route** |  |  |  |  | |
| MSM | 1418 (65.2%) | 2879 (61.5%) | 1195 (62.9%) | 5358 (68.6%) | |
| Heterosexual | 610 (28.0%) | 1501 (32.1%) | 560 (29.5%) | 1993 (25.5%) | |
| Intravenous drug use | 15 (0.7%) | 38 (0.8%) | 25 (1.3%) | 79 (1.0%) | |
| Blood-blood contact | 9 (0.4%) | 38 (0.8%) | 24 (1.3%) | 78 (1.0%) | |
| Vertical | 15 (0.7%) | 13 (0.3%) | 10 (0.5%) | 31 (0.4%) | |
| Unknown | 109 (5.0%) | 209 (4.5%) | 87 (4.6%) | 277 (3.5%) | |
| Missing | 2 | 8 | 1 | 12 | |
| **Prior ART use (median years, IQR)** | 7 (4-12) | 9 (6-14) | 7 (4-12) | 10 (6-15) | |
| **CD4+ T-cell count nadir (median cells/mm^3^, IQR)** | 280 (140-440) | 260 (140-380) | 250 (120-410) | 250 (130-380) | |
| **HIV-RNA zenith (median log_10_ c/mL, IQR)** | 5.0 (4.5-5.4) | 5.0 (4.5-5.4) | 5.1 (4.6-5.6) | 5.0 (4.5-5.5) | |
| **Prior AIDS diagnosis** | 466 (21.4%) | 884 (18.9%) | 446 (23.4%) | 1618 (20.7%) | |
| **CD4+T-cell count current (median cell/mm^3^, IQR)** | 710 (520-930) | 700 (520-914) | 688 (493-894) | 690 (520-900) | |
| **HIV-RNA 50-200c/mL (blip) at start follow-up** | 78 (3.6%) | 124 (2.6%) | 42 (2.2%) | 178 (2.3%) | |
| **Prior ART regimens (median, IQR)** | 1 (0-3) | 2 (1-3) | 1 (0-3) | 2 (1-3) | |
| **NRTI backbone** |  |  |  |  | |
| 3TC/ZDV | 0 (0%) | 0 (0%) | 2 (0.1%) | 0 (0%) | |
| ABC/3TC | 1469 (67.4%) | 242 (5.2%) | 1356 (71.3%) | 380 (4.9%) | |
| TDF/FTC | 356 (16.3%) | 1734 (37.0%) | 336 (17.7%) | 2533 (32.3%) | |
| TDF/3TC | 0 (0%) | 88 (1.9%) | 8 (0.4%) | 145 (1.9%) | |
| TAF/FTC | 334 (15.3%) | 2615 (55.8%) | 199 (10.5%) | 4764 (60.8%) | |
| TAF/3TC | 0 (0%) | 7 (0.1%) | 1 (0.1%) | 6 (0.1%) | |
| **Anchor drug class** |  |  |  |  | |
| INSTI | 2178 (100%) | 1814 (38.7%) | 1902 (100%) | 3400 (43.4%) | |
| NNRTI | 0 (0%) | 2415 (51.5%) | 0 (0%) | 3117 (39.8%) | |
| PI | 0 (0%) | 457 (9.8%) | 0 (0%) | 1311 (16.8%) | |
| **Comorbidities** |  |  |  |  | |
| Hepatitis B Ag positive | 92 (4.3%) | 262 (5.7%) | 72 (3.9%) | 402 (5.3%) | |
| Hepatitis C IgG positive | 202 (9.3%) | 390 (8.3%) | 196 (10.3%) | 788 (10.1%) | |
| Obesity | 174 (12.4%) | 517 (12.6%) | 215 (11.3%) | 921 (11.8%) | |
| Diabetes mellitus 2 | 65 (4.6%) | 164 (4.0%) | 82 (4.3%) | 323 (4.1%) | |
| Hypertension | 403 (28.8%) | 1151 (28.0%) | 590 (31.0%) | 2279 (29.1%) | |
| Chronic kidney disease | 102 (7.3%) | 235 (5.7%) | 210 (11.0%) | 546 (7.0%) | |
| Cardiovascular disease | 55 (3.9%) | 151 (3.7%) | 72 (3.8%) | 353 (4.5%) | |
| Stroke | 21 (1.5%) | 55 (1.3%) | 23 (1.2%) | 123 (1.6%) | |
| Non-AIDS malignancy | 43 (3.1%) | 123 (3.0%) | 54 (2.8%) | 286 (3.7%) | |
| Abbreviations: 3TC, lamivudine; ABC, abacavir; ART, antiretroviral therapy; DTG, dolutegravir; FTC, emtricitabine; INSTI, integrase strand transfer inhibitor; MSM, men who have sex with men; (N)NRTI, (non-)nucleoside reverse transcriptase inhibitor; PI, protease inhibitor; TDF, tenofovir disoproxil fumarate; TAF, tenofovir alafenamide; ZDV, zidovudine. | | | | |  |

| **Supplementary table 2. Viral blips at 1 year of follow up in cases on DTG/3TC and controls on triple-drug antiretroviral regimens who had undetectable plasma HIV-RNA <50 copies/mL or a viral blip <200 copies/mL at the start of the observation period.** | | | | | | | | | | | | | | | | | | |
| --- | --- | --- | --- | --- | --- | --- | --- | --- | --- | --- | --- | --- | --- | --- | --- | --- | --- | --- |
|  | **DTG-based ART** | | | | | | | | | **Non-DTG-based ART** | | | | | | | | |
|  | **DTG/3TC (cases)**  **Baseline pVL** | | | | **DTG-based ART (controls)**  **Baseline pVL** | | | | **DTG/3TC (cases)**  **Baseline pVL** | | | | | **Non-DTG-based ART (controls)**  **Baseline pVL** | | | | |
|  | 50-200 | | <50 | | 50-200 | | <50 | | 50-200 | | | <50 | | 50-200 | | <50 | | |
|  | N | % | N | % | N | **%** | N | % | N | | % | N | % | N | % | N | | % |
| Observation period ended before pVL was measured | 0 | 0 | 7 | 1.8 | 2 | 18.2 | 50 | 6.5 | 0 | | 0 | 10 | 3.7 | 1 | 20.0 | 32 | | 5.5 |
| All pVL ≤50 | 6 | 75.0 | 362 | 94.8 | 6 | 54.5 | 699 | 90.9 | 3 | | 100 | 266 | 92.7 | 3 | 60.0 | 527 | | 91.7 |
| Highest pVL 51-200 | 2 | 25.0 | 12 | 3.1 | 2 | 18.2 | 15 | 2.0 | 0 | | 0 | 8 | 2.8 | 1 | 20.0 | 8 | | 1.4 |
| Highest pVL ≥201 | 0 | 0 | 1 | 0.3 | 1 | 9.1 | 5 | 0.7 | 0 | | 0 | 3 | 1.0 | 0 | 0 | 8 | | 1.4 |
| Total | 8 | 100 | 382 | 100 | 11 | 100 | 769 | 100 | 3 | | 100 | 287 | 100 | 5 | 100 | 575 | | 100 |
| All pVL are in copies/mL  Abbreviations: 3TC, lamivudine; ART, antiretroviral therapy; DTG, dolutegravir; pVL, plasma viral load. | | | | | | | | | | | | | | | | |  |  |

| **Supplementary table 3. Weight change during follow up in cases on DTG/3TC and controls on triple-drug antiretroviral regimens.** | | | | | | | | | |
| --- | --- | --- | --- | --- | --- | --- | --- | --- | --- |
|  |  | Baseline weight^1^ | | Weight during follow-up | | Weight change during follow-up | | Weight change per year | |
|  | N | mean | SD | mean | SD | mean | SD | mean | SD |
| DTG-based DTG/3TC | 112 | 81.3 | 1.6 | 82.6 | 1.5 | 1.3 | 0.4 | 2.1 | 1.2 |
| DTG-based DTG/3TC (TDF stopped) | 16 | 79.5 | 4.5 | 81.9 | 4.0 | 2.5 | 1.2 | 4.2 | 1.6 |
| DTG-based controls | 340 | 80.6 | 0.8 | 81.6 | 0.9 | 0.9 | 0.2 | 0.9 | 0.3 |
| non-DTG-based DTG/3TC | 72 | 82.4 | 1.9 | 83.3 | 1.9 | 0.9 | 0.5 | 1.1 | 0.8 |
| non-DTG-based DTG/3TC (TDF stopped) | 50 | 78.7 | 2.6 | 81.4 | 2.5 | 2.7 | 0.6 | 3.0 | 0.8 |
| non-DTG-based controls | 263 | 82.1 | 1.1 | 82.4 | 1.1 | 0.3 | 0.3 | 0.2 | 0.4 |

Weight is in kilogram

^1^The median number of days the ‘baseline body weight’ was measured before the start of follow-up for this analysis was 118 days for DTG-based cases, 36 days for DTG-based controls, 89 days for non-DTG-based cases, and 130 days for non-DTG-based controls.

Abbreviations: 3TC, lamivudine; ART, antiretroviral therapy; DTG, dolutegravir; SD, standard deviation.
